# Mental health plans and policies across the WHO European region

**DOI:** 10.1017/gmh.2024.88

**Published:** 2024-11-18

**Authors:** Zoe Guerrero, Anna Kågström, Akmal Aliev, Hana Tomášková, Yongjie Yon, Ledia Lazeri, Marge Reinap, Cassie Redlich, Ana Maria Tijerino Inestroza, Jason Maurer, Petr Winkler

**Affiliations:** 1 Department of Public Mental Health, National Institute of Mental Health, Klecany, Czech Republic; 2 WHO Collaborating Center for Public Mental Health Research and Service Development; 3 World Health Organization Regional Office for Europe, Copenhagen, Denmark; 4 Health Service and Population Research Department; Institute of Psychiatry, Psychology and Neuroscience; King’s College London, United Kingdom

**Keywords:** mental health plans and policies, public mental health, Europe, Mental Health Atlas, Ment al Health Action Plan 2013–2030

## Abstract

Evidence is scarce in terms of tracking the progress of implementation of mental healthcare plans and policies (MHPPs) in Europe, we aimed to map and analyze the content of MHPPs across the WHO European region.

We collected data from the WHO Mental Health Atlas 2011, 2017 and 2020 to map the development of MHPPs in the region. We contacted 53 key informants from each country in the European region to triangulate the data from WHO Mental Health Atlases and to obtain access to the national mental health plans and policies. We analyzed the content of MHPPs against the four major objectives of the WHO Comprehensive Mental Health Action Plan, and we also focused on the specificity and measurability of their targets.

The number and proportion of countries which have their own MHPPs has increased from 30 (52%) to 43 (91%) between 2011 and 2020. MHPPs are generally in line with the WHO policy, aiming to strengthen care in the community, expand mental health promotion and illness prevention activities, improve quality of care, increase intersectoral collaboration, build workforce and system capacity, and improve adherence to human rights. However, specific, and measurable targets as well as a description of concrete steps, responsibilities and funding sources are mostly missing. They often contain very little information systems, evidence and research, and mostly lack information on evaluating the implementation of MHPPs.

Progress has been made in terms of the development of MHPPs in the WHO Europe. However, MHPPs are often lacking operationalization and appropriate data collection for evaluation. This is then reflected in missing evaluation plans, which in turn leads to lessons not being learned. To enhance the potential for knowledge generation and demonstration of impact, MHPPs should be more specific and contain measurable targets with allocated responsibilities and funding as well as evaluation plans.

## Impact statement

In the absence of information regarding the progress of implementation of mental health policies and plans at the European level, it is important to establish an evidence base in this regard. This study provides insight into the progress of the implementation of mental health plans and policies and allows us to map them against larger goals such as the WHO Comprehensive Mental Health Action Plan.

Our results indicate that notable progress has been made in the implementation of mental health policies and plans in the European region, specifically in areas such as deinstitutionalization and the inclusion of people with lived experience in decision-making. Nevertheless, several areas, such as information systems, evidence, and research for mental health, still require substantial development. Furthermore, the absence of a comprehensive evaluation of MHPP implementation is a significant concern, given that MHPPs are potent instruments for driving real-world change. This article provides an overview of recent achievements and offers a baseline for discussion of the next steps in the creation, implementation and evaluation of MHPPs across the European region, in accordance with WHO guidelines.

## Introduction

In 2013, WHO Member States adopted and responded to the 65th World Health Assembly resolution and the global Comprehensive WHO Mental Health Action Plan (2013–2020) by recognizing mental health as a growing public health priority as well as an integral dimension of universal health coverage and sustainable development. The global action plan has since been extended to 2030 (WHO 2021), and within the European region of 53 member states, the WHO European Framework for Action on Mental Health 2021–2025 was developed and endorsed (WHO Regional Office for Europe 2022). The framework was created partly in response to the impacts of the COVID-19 pandemic, and it sets out objectives for the WHO European region that are in line with objectives in the abovementioned WHO global action plan (WHO 2021a). Increasing access to community care, strengthening prevention and promotion, and improving adherence to the Convention on the Rights of People with Disabilities (UN General Assembly 2007) are among the main WHO mental health policy objectives for the European region.

Countries are increasingly launching national mental health plans, policies or strategies, however, their content has not been systematically analyzed, and it is unclear to what extent they are in line with the WHO policy as well as to what extent they are implemented. Implementation can be difficult if the plans and policies are not specific. Also, implementation can be halted due to the system-level barriers surrounding the infrastructure and logistics of service provision. Lack of resources and funding might further complicate implementation, especially in low- and middle-income countries (Zhou et al. 2018). Evaluation of the systematic determination of countries’ achievements related to the implementation of different components of MHPPs is crucial for understanding the progress of implementation and for the identification of barriers and facilitators. This is so lessons can be learned and more specifically that implementation strategies and levers can be better matched to potential barriers in order to improve the likelihood of successful implementation.

In the present study, we aimed to map and analyze evidence on the existence, implementation and evaluation of mental healthcare plans and policies in the European region, while also reviewing their content.

## Methods

### Data collection

We focused on assessing the evidence regarding the existence and content of all national and/or regional mental health policies and plans in the WHO European region (*n* = 53 countries). We included MHPPs on suicide prevention or neurological disorders such as dementia to align with the WHO European Framework of Action on Mental Health. We operationalized a mental health plan as a detailed scheme for implementing strategic actions in the area of promotion, prevention, treatment and rehabilitation related to mental health conditions, which allows for the implementation of visions, values, principles and objectives articulated in mental health policy. We understand mental health policy as an organized set of values, principles and objectives for improving mental health and reducing the burden of mental health conditions in a population. Ultimately, mental health policy defines a vision for future action (WHO 2021b). Implementation was defined as all the activities which are being carried out to complete the goals set out in MHPPs, and evaluation is understood as the activities done to understand how and to what extent implementation has been successful, an evaluation plan sets out the concrete methodologies used to achieve evaluation.

We extracted data from the WHO Mental Health Atlas 2011, 2017 and 2020 (WHO MHAs) to map the existence and development of MHPPs in the region. The WHO MHA has been released every 3 years since 2011 (2011, 2014, 2017, 2020), and it is based on responses of a key informant who provides national data on the state of mental healthcare and access to care based on predefined questions. We charted data from WHO MHAs on the existence and implementation of mental health policies and plans into a table, as we aimed to map the progression and emergence of mental health policies in each country over time. We provide the overall number of countries with an MHPP.

To triangulate the data from WHO MHAs as well as to collect MHPPs and evaluation plans we contacted key informants from all 53 countries in the WHO European region. Key informants were nominated by the team of the WHO Regional Advisor on Mental Health (LL) and they were either focal points for the WHO pan-European Mental Health Coalition and/or past national data coordinators of the WHO MHA. Most often key informants were lead coordinators of mental health plans and policy implementation at the governmental level (such as a Ministry of Health), and some were representatives of local service providers implementing the mental health plan or policy. Each informant was asked via e-mail to provide the national mental health policy or plan the corresponding evaluation plan (if existing/available), and any regional mental health policies or plans and corresponding evaluation plans. Reminders were sent once after three weeks of receiving the first e-mail invitation. We tabulated and summarized the responses from the key informants (see Table 1 in the Supplementary Material).

### Data extraction

We used the READ approach to carry out the content analysis of extracted data from the MHPPs gathered through the key informant survey; i.e. we created a data extraction sheet in Excel in order to allow for rigorous analysis of documents (Dalglish et al. 2020). Specifically, we extracted the following data: level of implementation (national or regional), key objectives, policy area (mental health, suicide prevention, dementia prevention), policy content including targets and indicators, years of implementation, theoretical framework of the MHPPs, methods, tools of data collection, outcomes. Data was extracted by one researcher (ZG) and then cross-checked by two researchers (AK, AA). Where it was possible data was analyzed in its original language, if this was not possible Google Translate was used.

We conducted the against the objectives of the WHO Comprehensive Mental Health Action Plan 2013–2030: (1) To strengthen effective leadership and governance for mental health, (2) To provide comprehensive, integrated and responsive mental health and social care services in community-based settings, (3) To implement strategies for the promotion and prevention of mental health, (4) To strengthen information systems, evidence and research for mental health (WHO 2021b). We further divided data into the content and the intended implementation section. Content describes and counts targets and indicators in MHPPs directly related to the achievement of the aforementioned WHO objectives. For example, an indicator or activity related to the establishment and support of an inter-ministerial committee would be presented under the WHO’s Objective 1 (see above). The implementation then describes the intended methods, processes and approaches taken to meet the targets established in a given MHPP. For example, an implementation approach where stakeholder collaboration is described would be presented under WHO Objective 1 (see above).

## Results

### Mental health policies or plans in the WHO European region 2011–2020

In 2011, 30 out of 52 (58%) WHO European region member states who responded to the WHO Atlas had reported having either, a mental health policy or plan. Subsequent editions of the WHO Atlas, however, asked only about plans. In 2014, after the launch of the WHO Comprehensive Mental Health Action Plan 2013–2020, the number of countries having a mental health plan increased to 34 out of 49 (69%). In 2017, 37 countries out of 48 (77%) had reported to have a mental health plan. Finally, in the latest edition from 2020, 43 out of 47 (91%) reported having a mental health plan.

Data about indicators and targets collected in the two latest editions of the Mental Health Atlas (2017 and 2020) show that in 2017, 30 out of the 37 (81%) countries that indicated having a mental health plan had established corresponding indicators or targets of success. In 2020, 34 out of 43 (79%) countries with a mental health plan had established such indicators or targets. The development of the existence of MHPPs is shown in Table 2 in the Supplementary Material, which demonstrates a steady increase in mental health plan presence between 2011 and 2020.

### Content analysis of mental health policies and plans across the WHO European region 2020

From the 53 key informants representing 53 countries in the region and invited to participate in the current research, 40 key informant surveys were collected. According to the key informant survey responses, 38 countries reported having a mental health policy or plan, 18 reported having a regional mental health policy or plan, 19 reported having a national evaluation of implementation (with 5 having a separate stand-alone evaluation plan and report), and 7 reported having an evaluation at a regional level (see Table 1 in Supplementary Material). For the purpose of this study, the United Kingdom has been divided into the countries of England, Northern Ireland, Scotland and Wales as their mental healthcare systems are mostly independent and have separate MHPPs. Out of these 40 informants surveys collected, 4 countries had a different response than the one they provided in the Mental Health Atlas 2020, with three having new MHPPs previously not reported in the Atlas and one no longer having an MHPP. This could be explained by the fact that data collection for our survey was done at a later stage than that for the Mental Health Atlas 2020 edition. Five countries reported that their MHPP has been either created or renewed recently (2021, or 2022). Five other countries have ended their mental health plan in 2020 or 2021. Finally, eight countries have developed a long-term mental health plan spanning 10 or more years.

Therefore, the following sections focus on content analysis of 38 MHPPs and 5 evaluation reports from the following countries: Albania, Andorra, Armenia, Austria, Azerbaijan, Belgium, Croatia, Czech Republic, Denmark, Estonia, Finland, France, Greece, Hungary, Ireland, Italy, Kazakhstan, Kyrgyzstan, Latvia, Malta, Netherlands, North Macedonia, Norway, Poland, Portugal, Republic of Moldova, Russian Federation, Slovakia, Slovenia, Spain, Sweden, Switzerland, Turkey, Ukraine, UK (England, Northern Ireland, Scotland), Uzbekistan (citations are provided in the Supplementary Material). For ease of reporting, we divided these countries into the following regional groupings according to the UN geoscheme for Europe (UN Statistics Division 2024): Eastern Europe, Northern Europe, Southern Europe, and Western Europe.

Based on MHPPs content analysis we divided countries according to their approach to the development of MHPPs. Most of the countries (26 out of 38) have employed a top-down approach, which means that MHPPs were created by a group of key stakeholders, usually at the ministerial/governmental level. The plans and policies are then envisaged to be implemented by local health authorities and service providers. 8 (out of 38, 4 from Northern Europe) countries have developed a bottom-up approach, where MHPPs are created and implemented solely by regional authorities and service providers. Finally, 4 (out of 38) countries have used a broad national strategy with larger targets and goals combined with local mental health plans which have elaborated regionally specific activities towards those larger goals.

All of the analyzed MHPPs include activities towards at least one of the WHO Comprehensive Mental Health Action Plan 2013–2030 objectives. A large majority of countries included activities towards objective 2 (To provide comprehensive, integrated and responsive mental health and social care services in community-based settings) and objective 3 (To implement strategies for the promotion and prevention of mental health). The number of countries (out of 38) whose plans cover targets directly related to the objectives of the WHO Comprehensive Mental Health Action Plan is as follows: objective 1–22, objective 2–35, objective 3–31, objective 4–17 (see Table 3 in the Supplementary Material). Below, we provide a more detailed analysis of the content of MHPPs with respect to each of these four objectives.

#### Objective 1: To strengthen effective leadership and governance for mental health

With respect to leadership and governance, 11 countries (7 from Eastern Europe, 2 from Northern Europe and 2 from Southern Europe) have had targets and activities aimed at promoting and strengthening intersectoral collaboration and communication both at the governmental and local levels. Six countries focused on support of service user movements and inclusion of people with lived experience in the creation and implementation of MHPPs. Three countries addressed the establishment of legislation, with one focusing specifically on human rights legislation. Finally, one country addressed the establishment of a mental health department at the Ministry of Health and a mental health service at the regional health directorates.

#### Objective 2: To provide comprehensive, integrated and responsive mental health and social care services in community-based settings

In terms of activities relating to objective 2, 22 countries described creating new community services or including new pathways for transition into community care (10 from the East, 6 from the North, 4 from the South and 2 from West Europe). Related to this, integration of mental health care into primary health care was mentioned as a key activity by 11 countries. The creation of services aiding in social inclusion (such as housing or employment support) was mentioned by 7 countries and the overall improvement of quality of care by 9. Relating to the improvement of care and resource planning, 9 countries’ MHPPs included capacity building and support of human resources financially or via training. Financing increase was also considered as part of resource planning by 2 countries. Finally, addressing human rights in mental health care was implemented as an activity by 9 countries, such activities included the implementation of training programs for healthcare professionals, the creation of national guidelines and/or changes in legislation.

#### Objective 3: To implement strategies for the promotion and prevention of mental health

Mental health promotion and prevention is reported as a key activity in MHPPs in 31 countries. Sixteen countries include activities geared towards increasing general population awareness of mental health and mental health disorders, however, no specific mechanisms are described. Ten countries describe in their MHPPs targets related to prevention such as early interventions or identification. Finally, seven countries mention the implementation of activities relating to suicide prevention (six from Northern Europe).

#### Objective 4: To strengthen information systems, evidence and research for mental health

Objective 4 is the least mentioned objective. Nonetheless, MHPPs included activities related to information and communication technologies (2), support of research in the field of mental health (3), creation of appropriate guidelines on data collection and research (2), and support in the creation and sustainability of appropriate monitoring systems (5, 2 from Southern Europe, 2 from Northern and 1 from Eastern Europe). Although many countries do not include this objective in their MHPPs, some do acknowledge its need by underlining the usage of the best methods and tools for all research and implementation activities.

### Intended implementation methods in MHPPs

#### Objective 1: To strengthen effective leadership and governance for mental health

Most countries report international law and policies as a motivation for the implementation of a mental health plan (24 out of 38). WHO action plans and Sustainable Development Goals are also frequently mentioned as the basis for initiating and launching MHPPs.

Costing of the activities included in the plans is usually done formally at a basal level, i.e., rough estimates are provided to allow for the planning of expenditure. Only one plan mentions evaluation of the cost-effectiveness of implementation.

Stakeholders involved in implementation vary across the countries. Half of the countries, however, reported some involvement, if not leadership by the Ministry of Health (19 out of 38 countries).

Eleven countries reported the involvement of other ministries in the creation and implementation of MHPPs as well. This often includes the Ministry of Social Affairs (some countries have a joint Ministry of Health and Social Affairs), the Ministry of Justice, the Ministry of Education, and even the Ministry of Interior and Ministry of Defense (education of the workforce, such as the police). Five countries report national health or mental health institutions as key stakeholders in the implementation of MHPPs. eight countries report that regional authorities including local health authorities and local service providers are the key action leaders. Some countries also included NGOs as key participants in implementation (4 out of 38). Five countries reported having a committee or a board of key stakeholders which have collaborated on leading the implementation; such committees include service users, academic institutions and selected ministries. Interestingly, 1 country also reported having a research council. Finally, two countries have appointed a national coordinator for mental health plan implementation.

Only 4 countries reported in their MHPPs on the networks of communication between key stakeholders involved in implementation. Most countries assigned specific activities to concrete stakeholders, such as the participation of respective ministries in inter-ministerial committees or the implementation of specific programmes (36 out of 38). Some countries reported on the required outcomes of communication such as the number of reports needed from meetings between key stakeholders. However, none of the plans set up a specific method of communication or frequency of communication. The process of collaboration between key stakeholders is also not described. Finally, service user organizations are frequently mentioned as key stakeholders, however, there is seldom a description of their specific support or a guarantee of their involvement in the implementation. Only 2 countries assigned them to specific target implementation.

In terms of the contents of intervention, the MHPPs of countries vary greatly. Most of the plans simply report the current epidemiological situation in the country and set up targets which define activities and stakeholders in charge of those activities (23 out of 38), such as the creation of a national anti-stigma campaign assigned to local NGOs. However, very few plans specify the concrete steps which will be taken to complete each activity (5 out of 38).

#### Objective 2: To provide comprehensive, integrated and responsive mental health and social care services in community-based settings

In terms of implementation, approaches and processes are rarely described within MHPPs. Five countries (out of 38, 3 from Eastern Europe, and 2 from Southern Europe) included training of the mental healthcare workforce as a strategy of implementation. This includes not only training but also collaboration with other professions such as social care, and the creation of multidisciplinary teams for example. Other countries focus on implementing activities related to social rehabilitation and the creation of teams and centers with the aim of better social inclusion of people with mental health conditions (5 out of 38). Finally, two countries have created a mental health plan specifically intended for emergencies, such as pandemics, largely motivated by the impacts of COVID-19.

#### Objective 3: To implement strategies for the promotion and prevention of mental health

While all countries focus on mental health in general as the main target of the mental health plan – this predominantly includes severe mental illnesses and common mental disorders such as depression and anxiety – 2 countries have separate plans for substance abuse prevention and treatment, and another 2 countries have separate plans for suicide prevention. Suicide prevention is often embedded in the general MHPPs though. Only 3 countries (out of 38, 2 from Northern Europe, and 1 from Western Europe) provided a separate mental health plan for child and adolescent mental health; however, child and adolescent mental health plans are often embedded within the larger MHPP.

Overall, it can be stated that the implementation of promotion and prevention activities is often included in the general targets of MHPPs, however, the specific steps that should be taken in order to complete such activities are presented in separate documents rather than in the main document of each MHPPs.

#### Objective 4: To strengthen information systems, evidence and research for mental health

Evaluation or monitoring is mentioned as a key activity by 4 countries (2 from Southern Europe, 1 each from Eastern and Northern Europe); however, the evaluation plan is usually not elaborated on. Only two mental health plans provide protocols for evaluation, such as qualitative interviews with key service providers or analysis of data from national registries. In terms of setting up indicators for the activities, a large proportion of countries set up indicators in the form of outputs rather than numerical outcomes (15 out of 38). This means, for example, stating that a report from a certain activity will be produced, or that community services will be strengthened. In this sense, indicators become general targets rather than measurable achievements. Five countries provide specific numeric targets or indicators such as reaching a specific number of bed reductions or new community centres. Four countries provide a more complex system in which there are actions and numeric indicators assigned to each target, which means that there are outputs to be finalized as well as specific numerical indicator changes to be observed for each target or activity. However, such indicators seldom touch upon hard-to-quantify outcomes in domains such as human rights or prevention and promotion. Finally, nine countries did not set up or report any indicators or targets to be met.

Evidence of the implementation of evaluation activities is also very scarce. Most often plans point towards mental health laws and larger WHO action plans as the motivation for the creation of a mental health plan (24 out of 38).

## Discussion

MHPPs are important tools to facilitate improvements in mental health practices that are both urgently needed and long overdue (Jenkins 2003). The increasing number of countries within the WHO European region aligning their MHPPs with WHO mental health policies to address current mental health challenges signifies promising progress. Existing European MHPPs declare to address diverse challenges, including the imperative of deinstitutionalization as a stepping stone towards human rights achievements in the mental healthcare sector, the mounting burden of mental disorders, the shortage of mental health professionals, and the necessity for intersectoral collaboration, which are all long known problems (Winkler et al. 2017; Aliev et al. 2021). Furthermore, MHPPs actively promote human rights and frequently engage individuals with mental health conditions as vital stakeholders in mental healthcare decision-making or the provision of mental health services. This is especially important since mental health care practices in the region have been often found poorly adherent to human rights as embedded in the Convention on Rights of People with Disabilities (Winkler et al. 2020; Høyer et al. 2022).

However, there is plenty of room for improvement. MHPPs often lack comprehensiveness and specificity, leaving them at risk of remaining aspirational rather than actionable. Costing of activities, when addressed, tends to rely on rough estimates, indicating a need for more precise planning. While it is understandable that concrete funding may not be reported in the plans themselves, it is important to prioritize funding transparency in reporting. The specific, well-defined steps required to ensure the effective implementation of these policies are mostly absent, suggesting a great opportunity for enhancement in this area. Another gap is the rare inclusion of provisions for the evaluation of the implementation of MHPPs. Numerous countries have established success indicators or targets; however, these are often framed as outputs rather than outcomes. This distinction makes it challenging to assess the extent of their achievement. Without clearly defined targets and tangible outcomes, the implementation of MHPPs may lack accurate guidance. This lack of evaluation transparency may also be attributed to the opacity of the methods and tools employed to assess MHPP implementation (Aliev et al. 2023). This shortage of information not only affects accountability but also limits the ability to thoroughly assess the actual impact of the policies and it also perpetuates a cycle of insufficient reporting and inadequate preparation of tools for evaluating MHPP implementation, which is crucial for their overall success and effectiveness. However, we could consider the WHO Mental Health Atlas a first stepping stone in reporting MHPP progress, as it provides an attempt at reporting transparently the information on MHPP implementation.

The content of mental health promotion programs in Europe suggests that mental health care systems have struggled to keep pace with the developments of the last half-century. Many MHPPs continue to stress the importance of strengthening community-based care, signalling that deinstitutionalization has not been achieved across the region. Furthermore, a vast majority of countries highlight the urgent need for substantial improvements in prevention and mental health promotion, indicating a current underdevelopment in this area. Most notably, it is striking that only two countries have explicitly articulated their intention to increase funding for mental health. This fact may be considered concerning, especially when all countries fall short of aligning their mental health expenses with the share of mental illnesses on the global burden of diseases.

Regarding the methods of implementation, one of the key aspects identified by our analysis is the division of countries according to their approach to involvement of stakeholders into three categories: top-down, bottom-up, and mixed. The top-down approach consists of a group of stakeholders usually at the highest governmental level coming together to create MHPPs. The positives of this approach are the improved chance of consistent implementation, and the subsequent ability to compare across contexts. The negatives are a possible low buy-in from local authorities and the provision of interventions that are not tailored to specific regional contexts – resulting in potentially ineffective allocation of resources. The bottom-up approach calls for a devolved system in which actors and stakeholders who are in the field lead the creation and implementation of MHPPs. This approach allows for high levels of buy-in, and high-quality local implementation. However, establishing a standardized quality of care nationally and evaluation at the national level may be difficult to achieve. Finally, very few countries use a mixed approach where there is a broad national strategy with larger targets and goals combined with local mental health plans which have elaborated regionally specific activities towards those larger goals. This approach allows for high levels of success in implementation provided buy-in is established at all levels.

Looking ahead, it is imperative to expand activities in three critical domains: effective leadership and governance, funding, as well as the strengthening of information systems, evidence, and research. The latter is particularly crucial, as it directly relates to the previously mentioned issue of a lack of comprehensive evaluation. Robust information systems, evidence, and research play pivotal roles in ensuring accurate data collection and the development of MHPPs. These systems also enable continuous learning from past experiences, guiding the evolution of MHPPs. This seems to be especially important in the context of central and eastern Europe, a region characterized by a lack of evaluation culture which in turn increases the risks of scarce resources being spent ineffectively.

## Limitations

There are several limitations to the current study, mostly pertaining to the method of data collection and possible data extraction biases. Firstly, although invitations were extended to all countries in the European region, we cannot entirely rule out that only countries with established MHPPs were more inclined to participate and submit documentation for content analysis. Similarly, we cannot rule out that a country with an MHPP may have been omitted from the WHO Atlas. Secondly, data extraction bias exists as a single researcher conducted all data extraction for content analysis. Thirdly, the use of Google Translator for translating MHPPs from their original languages may have resulted in the loss of certain nuances. Finally, the nature of the data allowed us to assess the current situation of MHPP implementation and evaluation but did not allow us to assess further barriers and facilitators.

Furthermore, limitations are inherent to the nature of implementation science related to policy and plan development. First, in the context of global human rights advancements and progressive reforms, longer-term policies can quickly become outdated, rendering the policies, plans, and routine monitoring and evaluation in this study potentially obsolete and not reflective of the current state of national progress. Additionally, the complexity of implementation planning and processes can be influenced by political, cultural, and capacity-related factors, making them challenging to capture accurately. This includes how to feasibly measure and gather information about the extent of policy implementation and the ’success’ of a process that may take up to a decade to fully realize. The question of accountability for this process also arises – without a mechanism for ensuring implementation quality, it becomes difficult to prioritize and report on monitoring and evaluation activities.

## Conclusions

Over the past two decades, progress has been achieved in the development of MHPPs within the European region, and MHPPs have been a vital tool to improve mental health practices in Europe. While there is promising progress, there is room for improvement. MHPPs would benefit from clearer reporting on concrete activities including actionable plans with defined key stakeholders or implementing bodies, as well as clear reporting within MHPPs on human and financial resource availability and allocation. Greater focus should also be put on the prevention and promotion of mental health, as this seems an area that is currently underdeveloped based on activities reported in MHPPs. The absence of a comprehensive evaluation of the implementation of activities defined in MHPPs is a significant concern, given that MHPPs are potent instruments for driving real-world change. These shortcomings make it difficult to assess the effectiveness of the implementation of MHPPs. European populations may bear the high costs of lessons not being learned unless efforts are made to address these critical areas and enhance the effectiveness of MHPPs. By addressing these gaps, MHPPs can become more successful in achieving positive mental health outcomes.

## Supporting information

Guerrero et al. supplementary material Guerrero et al. supplementary material

## Data Availability

No new data was generated or analyzed in this study.

## Supplementary material

The supplementary material for this article can be found at http://doi.org/10.1017/gmh.2024.88.
