# Supplementary material for: Mental health plans and policies across the WHO European region
Source: Glob Ment Health (Camb). 2024 Nov 18;11:e110. doi: 10.1017/gmh.2024.88 (PMC11588412; doi:10.1017/gmh.2024.88)
Supplement: Guerrero et al. supplementary material [file S2054425124000888sup001.docx]

# Appendix

Table 1: Key informant responses summary

| **Country** | **National MH policy/plan** | **Evaluation national level** | **Regional MH policy/plan** | **Evaluation regional level** |
| --- | --- | --- | --- | --- |
| Albania | Yes | No | No | No |
| Andorra | Yes | No | Yes | No |
| Armenia | Yes | No | No | No |
| Austria | Yes | Yes | Yes | Yes |
| Azerbaijan | Yes | No | No | No |
| Belarus |  |  |  |  |
| Belgium | Yes | Yes | Yes | Yes |
| Bosnia and Herzegovina |  |  |  |  |
| Bulgaria |  |  |  |  |
| Croatia | Yes | No | No | No |
| Cyprus |  |  |  |  |
| Czech Republic | Yes | No | Yes | No |
| Denmark | Yes | No | Yes | No |
| Estonia | Yes | No | No | No |
| Finland | Yes | Yes | No | No |
| France | Yes | No | Yes | No |
| Georgia |  |  |  |  |
| Germany |  |  |  |  |
| Greece | Yes | No | No | No |
| Hungary | Yes | No | Yes | Yes |
| Iceland |  |  |  |  |
| Ireland | Yes | Yes | Yes | No |
| Israel |  |  |  |  |
| Italy | Yes | Yes | Yes | Yes |
| Kazakhstan | Yes | No | No | No |
| Kyrgyzstan | Yes | No | No | No |
| Latvia | Yes | Yes | No | No |
| Lithuania |  |  |  |  |
| Luxembourg |  |  |  |  |
| Malta | Yes | Yes | Yes | No |
| Monaco |  |  |  |  |
| Montenegro |  |  |  |  |
| Netherlands | Yes | Yes | Yes | No |
| North Macedonia | Yes | No | No | No |
| Norway | Yes | Yes | No | No |
| Poland | Yes | Yes | Yes | No |
| Portugal | Yes | Yes | Yes | No |
| Republic of Moldova | Yes | Yes | No | No |
| Romania |  |  |  |  |
| Russian Federation | Yes | No | No | No |
| San Marino | No | No | No | No |
| Serbia |  |  |  |  |
| Slovakia | Yes | Yes | Yes | Yes |
| Slovenia | Yes | Yes | No | No |
| Spain | Yes | Yes | Yes | Yes |
| Sweden | Yes | No | Yes | No |
| Switzerland | Yes | Yes | Yes | Yes |
| Tajikistan | No | No | No | No |
| Turkey | Yes | No | No | No |
| Turkmenistan |  |  |  |  |
| Ukraine | Yes | No | No | No |
| UK- England | Yes | Yes | Yes | / |
| UK- Northern Ireland | Yes | Yes | / | / |
| UK- Scotland | Yes | Yes | / | / |
| UK- Wales |  |  |  |  |
| Uzbekistan | Yes | No | No | No |
| **TOTAL (yes answers)** | **38** | **19** | **18** | **7** |

Table 2: Progress in the development of national Mental health plans and policies according to creation WHO Mental Health Atlas 2011-2020

| **Country** | **2011** | **2014** | **2017** | **2020** |  | |
| --- | --- | --- | --- | --- | --- | --- |
| Albania |  |  |  |  | Not reported/ country profile unavailable |  |
| Andorra |  |  |  |  | Yes, there is a policy or plan |  |
| Armenia |  |  |  |  | No, there is no policy or plan |  |
| Austria |  |  |  |  | There is either a policy or plan but not both |  |
| Azerbaijan |  |  |  |  |  |  |
| Belarus |  |  |  |  |  |  |
| Belgium |  |  |  |  |  |  |
| Bosnia and Herzegovina |  |  |  |  |  |  |
| Bulgaria |  |  |  |  |  |  |
| Croatia |  |  |  |  |  |  |
| Cyprus |  |  |  |  |  |  |
| Czech Republic |  |  |  |  |  |  |
| Denmark |  |  |  |  |  |  |
| Esonia |  |  |  |  |  |  |
| Finland |  |  |  |  |  |  |
| France |  |  |  |  |  |  |
| Georgia |  |  |  |  |  |  |
| Germany |  |  |  |  |  |  |
| Greece |  |  |  |  |  |  |
| Hungary |  |  |  |  |  |  |
| Iceland |  |  |  |  |  |  |
| Ireland |  |  |  |  |  |  |
| Israel |  |  |  |  |  |  |
| Italy |  |  |  |  |  |  |
| Kazakhstan |  |  |  |  |  |  |
| Kyrgyzstan |  |  |  |  |  |  |
| Latvia |  |  |  |  |  |  |
| Lithuania |  |  |  |  |  |  |
| Luxembourg |  |  |  |  |  |  |
| Malta |  |  |  |  |  |  |
| Monaco |  |  |  |  |  |  |
| Montenegro |  |  |  |  |  |  |
| Netherlands |  |  |  |  |  |  |
| North Macedonia |  |  |  |  |  |  |
| Norway |  |  |  |  |  |  |
| Poland |  |  |  |  |  |  |
| Portugal |  |  |  |  |  |  |
| Republic of Moldova |  |  |  |  |  |  |
| Romania |  |  |  |  |  |  |
| Russian Federation |  |  |  |  |  |  |
| San Marino |  |  |  |  |  |  |
| Serbia |  |  |  |  |  |  |
| Slovakia |  |  |  |  |  |  |
| Slovenia |  |  |  |  |  |  |
| Spain |  |  |  |  |  |  |
| Sweden |  |  |  |  |  |  |
| Switzerland |  |  |  |  |  |  |
| Tajikistan |  |  |  |  |  |  |
| Turkey |  |  |  |  |  |  |
| Turkmenistan |  |  |  |  |  |  |
| Ukraine |  |  |  |  |  |  |
| UK |  |  |  |  |  |  |
| Uzbekistan |  |  |  |  |  |  |

Table 3: Content and intended implementation methods addressed in WHO Comprehensive Mental Health Action Plan according to country

| **Country** | **Obj 1** | **Obj 2** | **Obj 3** | **Obj 4** |
| --- | --- | --- | --- | --- |
| Albania | X | X | X |  |
| Andorra | X | X | X | X |
| Armenia |  | X | X |  |
| Austria |  | X | X |  |
| Azerbaijan | X | X | X |  |
| Belgium |  | X | X |  |
| Croatia | X | X | X | X |
| Czech Republic | X | X | X | X |
| Denmark |  | X | X | X |
| Estonia | X | X | X |  |
| Finland | X | X | X | X |
| France | X | X | X |  |
| Greece |  | X |  |  |
| Hungary |  | X |  |  |
| Ireland | X | X |  |  |
| Italy | X | X | X | X |
| Kazakhstan |  | X |  | X |
| Kyrgyzstan |  | X |  |  |
| Latvia |  | X | X |  |
| Malta | X | X | X |  |
| Netherlands | X | X | X | X |
| North Macedonia | X | X | X | X |
| Norway |  | X | X |  |
| Poland |  | X | X |  |
| Portugal | X | X | X | X |
| Republic of Moldova | X | X | X |  |
| Russian Federation | X | X |  |  |
| Slovakia | X | X | X |  |
| Slovenia | X | X | X | X |
| Spain | X | X | X | X |
| Sweden | X | X | X | X |
| Switzerland | X |  | X | X |
| Turkey |  | X | X | X |
| Ukraine | X | X | X | X |
| UK- England |  | X | X | X |
| UK- Northern Ireland |  |  | X |  |
| UK- Scotland |  |  | X |  |
| Uzbekistan |  | X |  |  |

Citations for received MHPPs

Albania

**Health Ministry, Republic of Albania** (2013) Action plan for development of mental health services in Albania 2013-2022. Available from: <https://shendetesia.gov.al/wp-content/uploads/2018/03/Plani_i_Vperimit_per_Zhvillimin_e_Sherbimeve_te_Shendetit_Mendor_2013_-_2022.pdf> (accessed 15 January 2023).

Andorra

Mental Health and Addictions Comprehensive Plan 2021-2030 (PISMA – Plan Integral de Salut Mental I Addiccions). Available through key informant personal communication.

Armenia

**Sakunts A, Temuryan M, Ghazaryan S** (2016) Implementation of 2014-2019 Strategy of Maintenance and Improvement of Mental Health in the Republic of Armenia. Available from: <https://hcav.am/wp-content/uploads/2020/07/ENG_mental-health-strategy-assessment_2014-2019.pdf> (accessed 15 January 2023).

Austria

**Arrouas M** (2019) Health goal 9. Psychosocial health promote all population groups. Supplemented working group report. Available from: <https://gesundheitsziele-oesterreich.at/website2017/wp-content/uploads/2019/03/bericht_gz9_ergaenzt.pdf> (accessed 15 January 2023).

Azerbaijan

**Mammadov J, Geraybeyli G** (2009) National mental health strategy. Available from: <http://www.psm.az/uploads/faydali_melumatlar/Psixi_saglamliq_sahesinde_milli_strategiya.pdf> (accessed 15 January 2023).

Belgium

Guide towards better mental health care through the creation of circuits and networks of needs (Brussel). Available from: <http://www.psy107.be/files/Bruxelles.pdf> (accessed 15 January 2023).

Croatia

National mental health protection strategy for the period from 2011 to 2016 (Croatia) (2010) Available from: <https://vlada.gov.hr/UserDocsImages/2016/Sjednice/Arhiva/79-4.pdf> (accessed 15 January 2023).

Czech Republic

**Ministry of Health Czech Republic** (2020) National action plan for mental health 2020-2030. Available from: <https://www.mzcr.cz/wp-content/uploads/2020/01/Národní-akční-plán-pro-duševní-zdraví-2020-2030.pdf> (accessed 15 January 2023).

Denmark

**Ministry of Health and the Elderly Denmark** (2018) We run together: A comprehensive action plan for psychiatry towards 2025. Available from: <https://sum.dk/Media/637643698522009991/Vi%20l%c3%b8fter%20i%20f%c3%a6llesskab%20-%20en%20samlet%20handlingsplan%20for%20psykiatrien%20frem%20mod%202025.pdf> (accessed 15 January 2023).

Estonia

**Social Ministry Estonia** (2020) Green paper on mental health. Available from: <https://www.sm.ee/sites/default/files/news-related-files/vaimse_tervise_roheline_raamat_0.pdf> (accessed 15 January 2023).

Finland

**Ministry of Social Affairs and Health Finland** (2020) National mental health strategy and programme for suicide prevention 2020-2030. Available from: <https://julkaisut.valtioneuvosto.fi/bitstream/handle/10024/162234/STM_2020_15.pdf?sequence=1&isAllowed=y> (accessed 15 January 2023).

France

**Ministry of Health France** (2018) French roadmap for Mental Health and Psychiatry. Available from: <https://sante.gouv.fr/prevention-en-sante/sante-mentale/Feuille-de-route-de-la-sante-mentale-et-de-la-psychiatrie-11179/> (accessed 15 January 2023).

Greece

**Working group of the Mental Health Sector, Institute of Scientific Research, Panhellenic Medical Association** (2021) Overall proposal for the Mental Health policy in Greece. Available through key informant personal communication.

Hungary

**Ministry of Human Resources** (2021) National Mental Health Program. Available from: <https://www.parlament.hu/irom41/16118/adatok/fejezetek/20.pdf> (accessed 15 January 2023).

Ireland

**Department of Health Ireland** (2019) Sharing the Vision: A Mental Health Policy for Everyone. Available from: <https://www.gov.ie/en/publication/2e46f-sharing-the-vision-a-mental-health-policy-for-everyone/> (accessed 15 January 2023).

Italy

Available through key informant personal communication.

Kazakhstan

**Ministry of Health Kazakshtan** (2017) Roadmap for the Development of the Mental Health Service of the Republic of Kazakhstan for 2017-2018. Available through key informant personal communication.

Kyrgyzstan

**Ministry of Health Kyrgyzstan** (2018) Action plan for 2018-2022. Available through key informant personal communication.

Latvia

Available through key informant personal communication.

Malta

**Ministry for Health Malta** (2019) A Mental Health Strategy

for Malta 2020-2030. Available from: <https://health.gov.mt/wp-content/uploads/2023/04/Building_Resilience_Transforming_Services_A_Mental_Health_Strategy_for_Malta_2020-2030_EN.pdf> (accessed 15 January 2023).

Netherlands

**Ministry of Health Netherlands** (2019) Mental health care outline agreement. Available through key informant personal communication.

North Macedonia

**Ministry of Health, Republic of North Macedonia** (2018) National Strategy for Promotion Mental Health in Republic of Macedonia September 2018-2025 with Action Plan (September 2018-2025). Available from: <https://zdravstvo.gov.mk/wp-content/uploads/2020/05/strategija-za-MZ-2018-2025-170718-pf-1.pdf> (accessed 15 January 2023).

Norway

**Research Council of Norway** (2009) Escalation Plan for Mental Health. Available from: <https://www.forskningsradet.no/siteassets/publikasjoner/1248431256883.pdf> (accessed 15 January 2023).

Poland

**Department of Health Poland** (2017) Implementation of tasks national program mental health protection. Available through key informant personal communication.

Portugal

**Technical Committee to Follow Mental Health Reform** (2017) National Mental Health Plan 2007-2016 and priority proposals for extension to 2020. Available through key informant personal communication.

Republic of Moldova

Available through key informant personal communication.

Russian Federation

Available through key informant personal communication

Slovakia

**Ministry of Health Slovakia** (2019) National program for Mental Health. Available through key informant personal communication.

Slovenia

**National Institute of Public Health Slovenia** (2020) National Mental Health Programme. Available from: <https://www.zadusevnozdravje.si/wp-content/uploads/2021/06/Mira_resolucija-ANG_splet-2020_FINAL.pdf> (accessed 15 January 2023).

Spain

**Ministry of Health Spain** (2021) Strategy Mental Health of the National Health System Period 2021-2026. Available through key informant personal communication.

Sweden

**National Coordinator in the Field of Mental Health Sweden** (2016) National Mental Health Strategy for 2016-2020. Available from: <https://docplayer.se/41324737-Fem-fokusomraden-fem-ar-framat.html> (accessed 15 January 2023).

Switzerland

**Swiss Confederation** (2016) Intended mental health measures in Switzerland, Report in fulfillment of the postulate of the Commission for Social Security and Health of the Council of States (SGK-SR) (13.3370) of May 3, 2013. Available through key informant personal communication.

Turkey

**Ministry of Health Turkey** (2020) National Mental Health Action Plan (2020-2023). Available through key informant personal communication.

Ukraine

Available through key informant personal communication.

UK / England

**National Health Service England** (2019) NHS Mental Health Implementation Plan 2019/20 – 2023/24. Available from: <https://www.longtermplan.nhs.uk/wp-content/uploads/2019/07/nhs-mental-health-implementation-plan-2019-20-2023-24.pdf> (accessed 15 January 2023).

UK / Northern Ireland

**Department of Health Northern Ireland** (2021) Mental Health Strategy 2021-2031. Available from: <https://www.health-ni.gov.uk/publications/mental-health-strategy-2021-2031> (accessed 15 January 2023).

UK / Scotland

**Scottish Government** (2018) Better Mental Health in Scotland. Available from: <https://www.gov.scot/binaries/content/documents/govscot/publications/strategy-plan/2018/12/programme-government-delivery-plan-mental-health/documents/better-mental-health-scotland/better-mental-health-scotland/govscot%3Adocument> (accessed 15 January 2023).

Uzbekistan

Available through key informant personal communication.

# 
