# Supplementary material for: Mental health plans and policies across the WHO European region
Source: Glob Ment Health (Camb). 2024 Nov 18;11:e110. doi: 10.1017/gmh.2024.88 (PMC11588412; doi:10.1017/gmh.2024.88)
Supplement: Peer review history

# Editor report: Recommendation: Mental health plans and policies across the WHO European region — R0/PR2

**DOI:** 10.1017/gmh.2024.88.pr2

**Affiliations:** Pravara Institute of Medical Sciences, India

One of the reviewers has mentioned that the conclusions are not grounded in the data presented. The reviewer also mentions the search is not systematic and comprehensive. These are major concerns and I would request authors to address all the reviewer comments.

# Editor report: Decision: Mental health plans and policies across the WHO European region — R0/PR3

**DOI:** 10.1017/gmh.2024.88.pr3

**Affiliations:** Johns Hopkins University Bloomberg School of Public Health, United States

No accompanying comment.

# Editor report: Recommendation: Mental health plans and policies across the WHO European region — R1/PR5

**DOI:** 10.1017/gmh.2024.88.pr5

**Affiliations:** Pravara Institute of Medical Sciences, India

No accompanying comment.

# Editor report: Decision: Mental health plans and policies across the WHO European region — R1/PR6

**DOI:** 10.1017/gmh.2024.88.pr6

**Affiliations:** Johns Hopkins University Bloomberg School of Public Health, United States

No accompanying comment.
